# Legitimation, transmission, and continuity: exploring the heart-of-mind esoteric Buddhist tradition in contemporary China

**DOI:** 10.3389/fsoc.2025.1701436

**Published:** 2025-11-12

**Authors:** Daohua Xu, Yaoping Liu

**Affiliations:** Department of Global Buddhism, Institute of Science Innovation and Culture, Rajamangala University of Technology Krungthep, Bangkok, Thailand

**Keywords:** Esoteric Buddhism, religious legitimacy, ritual transmission, contemporary Chinese Buddhism, Heart-of-Mind tradition

## Abstract

Existing research on legitimation has largely centered on historical and political narratives, overlooking the complex mechanisms through which contemporary practitioners construct, negotiate, and assert religious authority and authenticity. This study examines the processes of legitimation, transmission, and continuity within the *Esoteric Buddhist Tradition* in contemporary China. Drawing on 18 months of ethnographic fieldwork—including participant observation at three key sites (Yuanyin Temple, the ancient Yuanyin training site, and the Damodong Temple complex)—and 25 semi-structured interviews with monks, ritual specialists, and lay disciples, this research reveals that legitimacy is co-constructed through both internal and external forces. Internally, 84% of informants emphasized charismatic leadership and ritual efficacy as central to sustaining spiritual authority, while 72% highlighted the significance of lineage authenticity. Externally, institutional recognition from provincial Buddhist associations and participation in at least seven inter-sectarian forums between 2022 and 2024 provided crucial validation and public visibility. Furthermore, sustainability practices emerged as adaptive responses to contemporary challenges: 68% of sites integrated environmental ethics into temple management, 56% adopted digital media platforms for ritual dissemination, and 41% developed youth-oriented engagement programs. Collectively, these findings demonstrate that the legitimation, transmission, and continuity of the Esoteric Buddhist Tradition in modern China are dynamically constituted through the interplay between internal charisma, ritual authority, and institutional embeddedness within a rapidly modernizing socio-religious landscape.

## Introduction

The Esoteric Buddhist tradition in contemporary China denotes the revitalization and transformation of tantric or *Zhenyan* (True Word) practices—such as mandalas, mantras, mudrās, and deity yoga—initially transmitted during the Tang dynasty, which have been reintroduced in modern contexts through transnational exchanges and subsequently incorporated into established Chinese Buddhist institutions (Fan, 2024; Shen, 2024). Within this broader revival, the exploration of the Heart-of-Mind Esoteric Buddhist tradition highlights how esoteric practices are reinterpreted as inward, contemplative disciplines that emphasize the cultivation of mind and consciousness, thereby reshaping their role in the contemporary religious landscape of China (Lama, 2023).

Although recent scholarship has examined the revival of Esoteric Buddhism in China, existing studies often emphasize institutional reconstruction, cross-border transmission, or the influence of Japanese Shingon and Tibetan Vajrayāna, while paying insufficient attention to the specifically Chinese articulation of the Heart-of-Mind Esoteric Buddhist tradition (Jaeger, 2023). Academic discourse on Esoteric Buddhism in modern China tends to focus on two poles: preserving the classical Zhenyan heritage of the Tang Dynasty and adapting Tibetan Vajrayāna forms to the Han Chinese environment (Wu, 2024). Local traditions that evolved independently and are not directly connected to Japanese or Tibetan institutions, such as Heart-of-Mind, have seldom been the subject of academic inquiry (Rambo et al., 2012; Sullivan, 2025).

Research on legitimation has largely focused on historical or political narratives, overlooking the nuanced ways practitioners negotiate authority and authenticity in contemporary contexts (Bobichon, 2018; Tallberg and Zürn, 2019). Similarly, studies on transmission have prioritized external lineages and transnational flows, yet they rarely address how esoteric practices are localized, internalized, and adapted within the framework of Heart-of-Mind cultivation. In terms of continuity, prior works tend to highlight revival in institutional or ritual forms but have not adequately explored the experiential, contemplative, and philosophical dimensions that sustain this tradition in modern Chinese Buddhism (Jaeger, 2023; Zhang and Ji, 2018). This leaves a significant gap in understanding how legitimation, transmission, and continuity intersect in shaping the evolving identity of the Heart-of-Mind Esoteric Buddhist tradition in contemporary China (Bahir, 2021; Chen, 2024a,b). Studies of the relationship between hidden teachings and local practices in China show that the adaptation process of esoteric teachings is always influenced by historical context and cross-cultural interactions (Chen, 2025; Zhang, 2025). The research focuses on the interplay between legitimation, transmission, and continuity of Esoteric Buddhist Tradition in Contemporary China. This approach is expected to make conceptual contributions to studying religious discourse on Esoteric Buddhism in East Asia.

## Theoretical framework

Discourses on legitimacy in religious traditions are often grounded in Max Weber’s (1922/1978) tripartite typology of religious authority—traditional, charismatic, and rational-legal authority (Brown, 2023). Within the context of Esoteric Buddhism, these three dimensions intersect dynamically rather than existing as discrete categories. Traditional authority emerges through lineage transmission and ritual continuity, charismatic authority manifests in the personal magnetism and spiritual efficacy of the master, and rational-legal authority operates through institutional recognition and bureaucratic endorsement. Legitimacy, therefore, is not a static doctrinal principle but a socially negotiated process that is continuously reconstituted through ritual practice, textual production, and the circulation of symbolic capital among teachers, disciples, and institutions.

Empirical findings from participant observation and interviews in the Heart-of-Mind Esoteric Buddhist tradition reveal that 68% of monastic respondents emphasized ritual transmission and teacher-pupil relationships as the primary locus of traditional authority. Meanwhile, 84% of lay participants identified the charisma of the spiritual leader—his mastery of esoteric rituals and ability to mediate spiritual experiences—as central to legitimizing the community’s practices. At the same time, rational-legal authority is reinforced through institutional validation, as evidenced by the Heart-of-Mind tradition’s participation in seven provincial inter-sectarian forums (2022–2024) and partial recognition by local Buddhist associations. These empirical patterns illustrate how Weberian forms of authority converge to form a hybrid model of legitimacy, in which traditional lineage and charismatic embodiment are operationalized within an emerging bureaucratic framework of state-sanctioned religious regulation.

Earlier studies (Frydenlund, 2017; Zhang and Ji, 2018; Lama, 2023) similarly observed that modern Buddhist legitimacy in East Asia is shaped by hybridized and adaptive reinterpretations of inherited traditions rather than linear succession. In China, the legitimation of Esoteric Buddhism is further mediated by claims to transhistorical continuity—invoking Tang Dynasty affiliations, cross-cultural connections with Japan, and locally authenticated genealogies preserved in ritual documents. However, the Heart-of-Mind tradition departs from such genealogical claims. Instead, it constructs legitimacy through a localized historical narrative, referencing the revival of Qing-period ritual practices while adapting esoteric values to contemporary moral and environmental concerns. This aligns with Rambo et al.’s (2012) notion of “dynamic legitimacy,” where authority emerges through social resonance and contextual transformation rather than fixed institutional descent.

Integrating Weber’s typology into this framework highlights that the Heart-of-Mind tradition sustains its religious authority through a synergistic interplay: charismatic legitimacy provides immediate affective power; traditional legitimacy anchors authority in symbolic continuity; and rational-legal legitimacy ensures social endurance through institutional negotiation. Together, these dimensions explain how a minority esoteric lineage maintains vitality within a state-regulated religious environment, balancing ritual orthodoxy with digital engagement, environmental ethics, and youth participation. This synthesis underscores that legitimacy in contemporary Esoteric Buddhism is both relational and performative, continuously recalibrated between the demands of history, charisma, and institutional modernity.

This study employs Max Weber’s tripartite model of religious authority—charismatic, traditional, and rational-legal—as the core analytical lens to examine the legitimation, transmission, and continuity of the Heart-of-Mind Esoteric Buddhist tradition. Charismatic authority is operationalized through the analysis of the master’s spiritual charisma, ritual efficacy, and personal influence on disciples; traditional authority is traced through textual transmission, lineage continuity, and ritual pedagogy; while rational-legal authority is examined via institutional recognition, regulatory compliance, and bureaucratic legitimation by provincial Buddhist associations. These three analytical dimensions structure the coding and interpretation of ethnographic data—field notes, interviews, and ritual observations—allowing the study to reveal how legitimacy is constructed through the interplay of embodied charisma, inherited tradition, and formal institutional validation. By applying Weber’s framework, the research conceptualizes legitimacy not as a static attribute, but as a dynamic, multi-layered process of religious negotiation in contemporary China.

### Research of Esoteric Buddhism in global contexts

The study of Esoteric Buddhism in contemporary China has increasingly attracted scholarly attention, particularly in relation to its historical revival, cross-cultural exchanges, and institutional reconstruction. Yet much of the existing literature remains limited in scope, often privileging political legitimation, transnational lineages, or organizational continuity while overlooking the nuanced ways practitioners negotiate authority, adapt transmission processes, and sustain contemplative traditions in modern contexts.

First, previous scholarship has examined the legitimation of Buddhist traditions in China primarily through historical and political frameworks. Hammerstrom (2013) emphasizes how religious legitimacy has often been linked to dynastic patronage and political recognition, while Davidson (2023) highlight the role of institutional authority in shaping discourses of authenticity. Although these perspectives underscore the importance of external validation, they tend to understate the agency of practitioners in negotiating legitimacy within lived religious contexts (Orzech et al., 2011). This leaves unresolved questions regarding how charisma, ritual efficacy, and grassroots religious authority function as parallel or complementary modes of legitimation in the contemporary revival of Esoteric Buddhism, particularly in the Heart-of-Mind tradition.

Second, research on the transmission of Esoteric Buddhism has tended to prioritize external lineages, such as the influence of Tibetan Vajrayāna or Japanese Shingon, with attention focused on transnational flows of teachings and ritual manuals. While these studies illuminate the global dimensions of esoteric transmission, they often overlook the processes by which esoteric practices are localized and internalized by Chinese practitioners. Existing works rarely explore how ritual forms are adapted into the framework of Heart-of-Mind cultivation, where emphasis is placed on inner transformation and meditative praxis. This omission has limited scholarly understanding of the creative negotiations through which esoteric knowledge is transmitted in a way that resonates with local spiritual sensibilities and contemporary concerns (An, 2023; Wenta, 2023).

Third, in terms of continuity, studies have frequently emphasized institutional revivals and the restoration of ritual practices as markers of the endurance of Esoteric Buddhism. Jaeger (2023) discusses the role of Buddhist associations in maintaining esoteric rituals, while Zhang and Ji (2018) analyze the reconstruction of liturgical frameworks. Yet such institutional approaches risk reducing continuity to organizational survival, neglecting the experiential, contemplative, and philosophical dimensions that animate esoteric traditions in lived practice (Weber, 2013). As a result, the enduring role of inner cultivation, ethical reinterpretations, and philosophical engagement in sustaining the Heart-of-Mind tradition remains underexplored in the broader discourse on Chinese Buddhist modernity.

Finally, recent studies on hidden teachings and local practices suggest that the adaptation of esoteric traditions in China is shaped not only by historical precedents but also by ongoing cross-cultural exchanges (Grundmann, 2021; Orzech et al., 2011; Wu, 2024). Wu (2024) further underscore the need to examine the intersection of legitimation, transmission, and continuity in shaping evolving religious identities. These findings imply that the Heart-of-Mind tradition cannot be understood solely through one dimension, but rather through the interplay of authority construction, localized adaptation, and experiential sustainability. By focusing on these intersections, future research may contribute conceptually to broader discussions of religious discourse and the transformation of Esoteric Buddhism within the East Asian context (Orzech et al., 2011).

Based on the identified gap, the following research questions are then formulated as follows:

1. How do practitioners and institutions of the Heart-of-Mind Esoteric Buddhist tradition in contemporary China construct its legitimation?
2. In what ways are esoteric practices of the Heart-of-Mind tradition transmitted across generational in modern Chinese society?
3. How is the continuity of the Heart-of-Mind Esoteric Buddhist tradition maintained its engagement with contemporary issues such as environmental ethics, digital engagement, and youth participation?

## Methodology

This research employs a qualitative-ethnographic approach with a reflective orientation, deemed most appropriate for uncovering the legitimacy, transmission, and sustainability dynamics of the Heart-of-Mind tradition in contemporary China. This approach was chosen based on the research objective of understanding the practices, narratives, and socio-religious structures from practitioners’ perspectives, while interpreting their meaning within a broader socio-political context.

Primary data were collected through continuous field observations over 3 years (2022–2024), with an intensive focus on the final year (2024). Observations were conducted at three key sites representing different functions within the spiritual structure of this tradition: the Yuanyin Temple in Jiangsu Province as the centre of legitimacy, the Yuanyin Ancient Ritual Training Site as a place for the preservation of rites, and the Damodong Temple Complex as a centre for intergenerational transmission and community expansion.

A total of 25 informants (see Table 1) were interviewed face-to-face using a semi-structured interview format. The sample comprised seven senior monks, six ritual teachers (*fashi*), eight lay community coordinators, and four local scholars engaged in research on contemporary Buddhism. Informants were selected through theoretical sampling, aimed at capturing participants who play pivotal roles in the interpretation, preservation, and negotiation of legitimacy within the Heart-of-Mind (*Xinfa*) Esoteric Buddhist network. This sampling logic followed the principle of maximum variation, ensuring representation across different hierarchical positions and ritual functions to reflect the structural diversity of the *Xinfa Tantra* community. The final sample size of 25 was deemed sufficient to achieve data saturation, as no substantially new themes emerged after the 23rd interview.

**Table 1:** Research participant.

Initial name	Gender	Age	Participant category	Experience
R01	Male	69	Senior monastic	27 years in esoteric Buddhist tradition
R02	Male	57	Senior monastic	29 years in esoteric Buddhist tradition
R03	Female	50	Senior monastic	17 years in esoteric Buddhist tradition
R04	Male	51	Senior monastic	26 years in esoteric Buddhist tradition
R05	Female	46	Senior monastic	18 years in esoteric Buddhist tradition
R06	Female	37	Junior monastic	3 years in monastic esoteric practice
R07	Female	40	Junior monastic	5 years in monastic esoteric practice
R08	Female	28	Junior monastic	10 years in monastic esoteric practice
R09	Female	35	Junior monastic	8 years in monastic esoteric practice
R10	Male	29	Junior Monastic	4 years in monastic esoteric practice
R11	Male	45	Lay practitioner	6 years practicing Heart-of-Mind
R12	Female	38	Lay practitioner	10 years practicing Heart-of-Mind
R13	Female	45	Lay practitioner	8 years practicing Heart-of-Mind
R14	Male	41	Lay practitioner	5 years practicing Heart-of-Mind
R15	Male	43	Lay practitioner	14 years practicing Heart-of-Mind
R16	Female	34	Lay practitioner	15 years practicing Heart-of-Mind
R17	Female	51	Lay practitioner	10 years practicing Heart-of-Mind
R18	Female	36	Scholar/researcher	Published research related to esoteric Buddhism
R19	Male	60	Scholar/researcher	Published research related to esoteric Buddhism
R20	Male	40	Scholar/researcher	Published research related to esoteric Buddhism
R21	Female	36	Community leader	Over 8 years organizing community activities
R22	Male	50	Community leader	Over 5 years organizing community activities
R23	Female	53	Community leader	Over 8 years organizing community activities
R24	Male	35	Community leader	Over 6 years organizing community activities
R25	Female	35	Community leader	Over 3 years organizing community activities

Interviews were conducted in Mandarin Chinese across three primary field sites—Yuanyin Temple (Jiangsu Province), the historical Yuanyin training site (Zhejiang Province), and the Damodong Temple complex (Anhui Province)—representing the main regional nodes of the *Xinfa Tantra* network in eastern China. While this geographical distribution captures the core operational sphere of the Heart-of-Mind lineage, it also presents a **regional limitation**, as the findings may not fully generalize to *Xinfa*-affiliated communities in northern or southwestern provinces. All interviews were audio-recorded, transcribed verbatim, and thematically analyzed to identify convergences and divergences in the construction and transmission of religious legitimacy.

In addition to interviews, this study utilized documentary and artifactual data, including liturgical manuscripts (jiben), master-disciple transmission records, recordings of blessing ceremonies, photographs and posters of community activities, and public statements from local Buddhist associations. The researchers also obtained permission to attend closed ritual sessions at two monasteries, including one initiation ceremony that was not open to the public. These observations enriched the verbal data with performative and visual dimensions.

Ethnographic data collection in studying the *Heart-of-Mind Esoteric Buddhist* tradition in contemporary China involves immersive fieldwork that captures the lived experiences, daily rituals, and training activities of practitioners. Researchers typically employ participant observation—joining meditation sessions, attending chanting ceremonies, and recording ritual performances—to understand how symbolic meanings are enacted and legitimized within institutional settings. For instance, observing how temple masters conduct *empowerment rituals* or how lay disciples engage in mindfulness exercises provides insight into how legitimacy is constructed through embodied practices and hierarchical authority. To control for researcher bias, reflexive journaling and triangulation are applied—researchers continuously assess how their positionality, expectations, and cultural assumptions might shape interpretations, ensuring that observations remain faithful to participants’ perspectives.

The transmission of *Heart-of-Mind* esoteric practices across generations requires close ethnographic attention to pedagogical processes, mentor-disciple relationships, and knowledge exchange mechanisms. Interviews with masters and apprentices, along with longitudinal observation of training routines and initiation rites, allow researchers to document how esoteric knowledge is ritualized, memorized, and reinterpreted in modern contexts. For instance, audio-visual documentation of oral instructions and digital archives of scriptures reveal how traditional teachings are translated for younger practitioners using multimedia tools. Controlling bias in such contexts involves comparative data collection—verifying accounts across multiple temples and age groups—and maintaining cultural humility to avoid privileging modern or Western interpretations of transmission and authenticity.

Finally, maintaining the continuity of the *Heart-of-Mind* Esoteric Buddhist tradition amid contemporary issues such as environmental ethics, digital engagement, and youth participation requires ethnographers to track institutional adaptations and value negotiations. Data collection may include observing temple-led ecological rituals, online meditation sessions, and youth-centered workshops that blend ancient mantras with sustainability discourse or social media outreach. Focus group discussions and digital ethnography techniques (e.g., following WeChat temple communities) can reveal how beliefs evolve within globalized and ecological narratives. Researchers manage potential bias by applying *member checking*—sharing interpretations with practitioners for validation—and by incorporating both digital and offline ethnographic evidence to ensure a holistic, contextually grounded understanding of how the tradition remains vibrant in modern Chinese society. The samples of interview guides are (a) Legitimacy of the Heart-of-Mind Tradition such as “How did the Heart-of-Mind Tradition gain legitimacy in the Buddhist community in China? (b) Promotion, such as “Who are the primary individuals engaged in the dissemination of this tradition?” and (c) Influence across generations, such as “How have the practices in this tradition changed over generations?”

The data analysis process was conducted through thematic analysis to organize and code the data. Each principal topic was discerned through a hermeneutic analysis that took into account the informants’ political stances, the intricacies of cultural censorship, and the historical backdrop of the accounts presented. A triangulation strategy was used to check the consistency of findings between interview data, direct observations, internal documents, and official publications such as the Buddhist Association of China report (2021).

From an ethical perspective, all informants fully explained the study’s purpose and consented to their participation through informed consent. In order to safeguard the confidentiality of informants, personal identities and sensitive information were concealed. The monastery administration and local religious authorities granted official permission for access to the research sites. Researchers are aware of their position as outside observers who have limitations in understanding certain internal meanings, so critical reflection on this position is carried out throughout the research process to minimize interpretive bias.

## Result

### Research question (RQ1): How do practitioners and institutions of the Heart-of-Mind Esoteric Buddhist tradition in contemporary China construct its legitimation?

The esoteric Heart-of-Mind Tradition (Xin-Yi Mijiao) derives its legitimacy through the transmission of spiritual lineages and the complex production of authority, combining historical narratives, symbolic rituals, institutional recognition, and spiritual performativity. Legitimacy in this context is not fixed, but is actively constructed and negotiated amidst interactions between religious actors, doctrinal texts, state institutions, and the lay public.

Max Weber’s tripartite framework categorizes religious legitimacy into three types: traditional; charismatic; and legal-rational. This framework theoretically enhances the legitimacy analysis in this study. These three forms are intertwined in Heart-of-Mind practices, creating a dynamic and adaptive pattern of legitimacy. Furthermore, there have been comparative attempts between Heart-of-Mind and orthodox Esoteric Buddhism (such as Zhenyan and Shingon), particularly in terms of doctrinal justification, lineage transmission, ritual manifestations, and community structure. These comparisons illustrate that Heart-of-Mind is not merely a local variant, but a contemporary adaptation that preserves the fundamental components of Mahāvairocana esotericism.

1. Doctrinal justification: sacred texts and esoteric authenticity

One of the main pillars of the Heart-of-Mind Tradition’s legitimacy is its adherence to a distinctive doctrinal authority aligned with orthodox esoteric foundations. This tradition considers the Buddha Heart Sutra (佛心經) its primary text, not only liturgical but also epistemological and performative. Its position parallels the Mahāvairocana Tantra in Shingon and the Hevajra Tantra in Vajrayāna, as a central text that serves as a medium for actualising Dharmakāya wisdom.

This research found that this doctrinal justification was established through an internal reading of the Heart Sutra Internal Commentary (佛心經內義疏), a manuscript used exclusively among senior monks. On the second page of the manuscript, it is written:

「佛心乃大日法身智慧之體, 非止八千偈語所及。傳習於大無外密傳, 非為外典, 而是本密所授。」.

*“Buddha-Heart is the embodiment of Mahāvairocana’s Dharmakāya-wisdom; it transcends the scope of the eight-thousand gāthās. It is transmitted not via exoteric scriptures but through the inner-most esoteric lineage.”*

This text asserts that the Buddha Heart Sutra is not simply a collection of verses, but a form of spiritual vibration that connects practitioners with the transcendent aspect of Mahāvairocana. Oral teachings reinforce this within the community by stating that the Buddha Heart Sutra is not to be recited but to be “resonantly felt.” A spiritual heir of Master Yuan Yin stated:

「在我們的傳統裏, 誦持佛心經不是念一句經, 而是讓法身的頻率在心中振動。」.

*“In our tradition, reciting the Heart Sutra is not uttering a verse; it is allowing the frequency of Dharmakāya to resonate within one’s heart.”*

This statement emphasizes that doctrinal legitimacy is not literalistic, but performative—where mantras and sutras are not mere recitations but are ontological acts that open up a direct connection with absolute reality (真如界, realm of suchness).

Within the framework of comparison, researchers highlight three dimensions that constitute the doctrinal strength of Heart-of-Mind: (1) Text as a cosmic bridge: The Heart Sutra is positioned, like the Mahāvairocana Tantra, not as information, but as a medium for spiritual actualizationactualizationactualization; (2) Mantra as a metaphysical frequency: Practice does not stop at sound, but emphasizesemphasizesemphasizes esoteric vibrations that activate the highest meditative states; (3) Inner transmission as the key to authority: This tradition does not employ formal abhiṣeka but upholds the practice of the “three secrets” (body, speech, and mind) transmitted orally in a closed circle, reflecting an orthodox esoteric structure.

Fieldwork also shows that this text is central to a confirmation ritual only after long-term meditation training. This ritual includes reciting secret mantras, contemplation of Buddha-Heart iconography, and focusing on the middle chakra (zhongxin 中心), believed to be the “inner door” to Dharmakāya resonance. Conceptually, this legitimating structure asserts that Heart-of-Mind does not establish a new orthodoxy but claims a position within a historical and mystical lineage equal to other Eastern esoteric traditions. Doctrinal justification becomes a kind of “spiritual inheritance” (靈脈繼承) that transmits the Mahāvairocana teachings to contemporary generations through a local Chinese path based on direct experience (體證) and inner resonance (感應). In this way, doctrine and ritual in Heart-of-Mind are not mere religious formalisms, but become a multi-layered strategy of spiritual authority: sacred texts, contemplative experience, and community structures all support each other in a living system of legitimacy.

2. Genealogy and historical narrative as symbolic foundations

The affirmation of legitimacy within the Heart-of-Mind tradition rests on a narrative construction that emphasizes the continuity of spiritual lineages dating back to the Qing Dynasty. A key internal document, the Silk Sutra of Continuity, states:

“自普陀山上大日如來傳法之始, 歷代祖師親授親承, 綿延不絕, 如燈續火, 我宗正是此續焰之一支。” (page. 3).

“Since Mahāvairocana planted the Dharma on Mount Putuo, the patriarchs of each generation have passed on the teachings directly and continuously, like one lamp lighting the next. Our tradition is one of the sparks of that flame.”

This quote symbolically positions Heart-of-Mind as an authentic part of mainstream Chinese Buddhism. The lineage is not simply a historical record, but a mythological construct used to establish claims to doctrinal authority and spiritual legitimacy. By placing itself within the Mahāvairocana Dharma lineage and the Chan School, Heart-of-Mind repositions itself from a fringe sect to the legitimate inheritor of the great esoteric teachings. This strategy demonstrates how historical narratives are mobilized as effective instruments of legitimacy, particularly in a state-controlled religious landscape sensitive to issues of doctrinal authenticity.

3. Ritual practice as performative legitimacy

Esoteric rituals in the Heart-of-Mind tradition serve as performative means of establishing and maintaining legitimacy. Observations at Yuanyin and Damodong Temples demonstrate that empowerment ceremonies (加持灌頂) are not only spiritual in nature but also create publicly recognizable structures of authority. When a disciple receives direct initiation from the teacher through secret mantras (密言), mudras (手印), and dharma symbols (法輪), what occurs is not simply a transfer of teachings, but a relational affirmation of the lineage’s legitimacy.

This is emphasized in the ritual text 真言行法要義 (Shingon Xingfa Yaoyi):

“當導師之手觸頂並耳誦密言, 即為有形與無形世界搭橋, 是認證, 非僅傳授。” (Chapter 5).

*“When the teacher’s hand touches the top of the pupil and the mantra is whispered, a bridge is formed between the world of form and formlessness. This is validation, not just teaching.”*

This ritual serves as a collective confirmation of one’s spiritual status within the transmission network, and makes the guru’s body a symbol of direct authority.

4. Institutional support and state symbols

Heart-of-Mind’s legitimacy is strengthened through strategic integration with official religious institutions in China. An official report from the Chinese Buddhist Association (CBA, 2021) states that the tradition has held a national training for esoteric teachers on “Social Ethics and the Closed Dharma” at Yuanyin Temple, which serves as a model for the compatibility of esoteric doctrine with the values of Chinese socialism.

The report states:

“心意密宗展現了一種順從、包容、貢獻社會主義核心價值的佛法實踐樣本。” (CBA, 2021, page. 17).

“Heart-of-Mind showcases Dharma practice that is obedient, inclusive, and contributes to the core values of socialism.”

This adjustment to the national narrative becomes an important instrument for gaining formal recognition and expanding institutional space for maneuver within the state-controlled system.

In addition to structural legitimacy, the individual charisma of leaders also plays a crucial role. Figures like Master LZ are revered across traditions, including Chan and Pure Land monasteries, as symbols of wisdom that transcends sectarian boundaries. R01 (a senior monk) stated:

“The existence of teachers who are respected by both Chan monasteries and Pure Lands is proof that we are not a deviant sect, but a bridge between traditions.”

In his internal biography it is stated:

“他不只是誦經者, 而是經文的化身。人們前來, 不只是為了法, 而是為了他的在場。” (*LZ Biography*, page. 4).

*“He is not just a sutra reader, but an incarnation of the sutra itself. People come not just for the teachings, but for his presence.”*

This charisma produces legitimacy that does not depend on institutions, but rather on personal resonance with the spiritual and social community.

5. Adaptation to contemporary social values

The Heart-of-Mind tradition has successfully reached a young, urban audience by adapting esoteric teachings into a contextual format. The “digital mindfulness” and “rites in the internet age” workshops provide innovative platforms for articulating the values of meditation, digital ethics, and social awareness in a modern context. This is not simply a technical strategy, but a transformation of the teachings’ meaning.

R20 (academic) said:

*“The transformation of the legitimacy of the Heart-of-Mind is not by rejecting the times, but by interpreting them from within the mantra.”*

This process demonstrates how legitimacy is built through relevance—not just fidelity to texts, but also through the capacity to respond to social change. Thus, Heart-of-Mind reconstructs esotericism not as a closed, ancient practice, but as an open, inclusive, and modern contemplative space.

### Research question (RQ2): In what ways are esoteric practices of the Heart-of-Mind tradition transmitted across generational in modern Chinese society?

The survival and expansion of the Heart-of-Mind Tradition depends heavily on central figures who act as promoters: teachers, abbots, and community organizers who not only maintain spiritual transmission but also bridge esoteric teachings with the dynamics of contemporary society. Fieldwork shows that these promoters are not merely inheritors but also innovators, shaping new transmission patterns and expanding socio-traditional networks through participatory and digital approaches.

The Heart-of-Mind tradition did not develop through a formal hierarchical structure, but rather through the presence of charismatic figures who catalyzed the regeneration and expansion of the teachings. The two main figures most frequently mentioned in interviews were Master LZ (罗志法师) and Abbot QW (清伟上人). These two figures represent two forms of authority: spiritual-transcendental and institutional-dialogical.

Master LZ is known as a “life mantra bearer” (活持咒者), as described by his senior student R04:

*“He is not a teacher. He is the guardian of the flame. He does not give answers, but lets you see how mantra lives through him.”*

Without formal Buddhist training, Master LZ developed the Sanri Rushi (3 days of entry into reality) retreat system, which combines Mahāvairocana Tantra recitation, closed meditation, and open Dharma dialogue. In his book Jingshen Chuanjie (精神傳接, 2019), he writes:

“師者非知識的搬運工, 而是把密咒交給靈魂的人.”

*“A master is not a transporter of knowledge, but one who delivers esoteric mantra to the soul.”*

In contrast, Abbot QW plays a more formal role in the realm of representation. He regularly attends Provincial Buddhist Association forums and represents Heart-of-Mind in intertraditional dialogues. His expertise lies in spiritual diplomacy and reframing esoteric narratives to make them compatible with public discourse.

Furthermore, unlike the vertical line patterns of Shingon or Vajrayāna, Heart-of-Mind adheres to a system of collective mentoring (jiti daoshi zhi 集體導師制). In this system, the promoter is not only responsible for leading the rites, but also for building the community and reproducing the teachings in a contemporary format. R06, a young teacher, explains:

“我們不只是學咒語。我們學怎麼管理道場, 怎麼設計課程, 怎麼拍影片, 也學怎麼辦一場好的祈福會。.”

*“We do not just learn mantras. We learn how to manage a temple, design courses, shoot videos, and organizeorganizeorganize a meaningful blessing event.”*

This model democratizesdemocratizesdemocratizes spiritual authority, creating functional roles such as retreat teacher, teaching content editor, festival coordinator, and digital media manager. One practice noted by researchers is a monthly reporting ritual in which each teacher keeps a journal of daily practice and files documentation of activities for review at district meetings.

Heart-of-Mind promoters actively transmit their teachings through digital channels such as WeChat, Bilibili, and YouTube. Fashi ZH’s online class “Mantra and a Balanced Life,” for example, has attracted participants from Chinese diaspora communities in Vancouver, Penang, and Sydney. Their digital posters often feature the Mahāvairocana symbol with modern visuals—such as esoteric calligraphy combined with cinematic elements. Their popular slogan:

“法不是死的文字, 而是呼吸著的心音。.”

*“The Dharma is not dead text, but the breath of the heart made audible.”*

This digitalization confirms that Heart-of-Mind does not view esotericism as an exclusive teaching, but rather as a living tradition that can be represented visually, communicatively, and contextually.

The presence of promoters is also evident in intersect interactions. Master LZ was once invited to be a guest speaker at a Chan retreat in Fujian Province and at an intersect Vesak celebration in Yunnan. Buddhist scholar R20 refers to this as “trans-traditional charisma”:

“他們雖然不是主流, 但講話讓所有人沉默。他的出現是跨宗的合法性。.”

*“They may not be mainstream, but their words silence the room. His presence constitutes trans-sectarian legitimacy.”*

This fact shows that promoters not only carry teachings, but also bridge social spaces: between mainstream and fringe, between conservative and progressive, between ritual and technology.

### Research question (RQ3): How is the continuity of the Heart-of-Mind Esoteric Buddhist tradition maintained its engagement with contemporary issues?

The sustainability of the Heart-of-Mind Tradition does not rest solely on lineage conservation or ritual preservation. It is the result of a complex orchestration of cadre regeneration, cultural adaptation, and legal engagement within the state-sanctioned framework of Chinese institutional Buddhism. Fieldwork indicates that this sustainability is strategically constructed through: (1) intergenerational spiritual education, (2) repackaging esoteric values into socially contextualized formats, and (3) formal collaboration with state-recognized religious structures.

1. Spiritual regeneration and an inclusive education model

Rather than relying on a single, authoritative transmission model from teacher to student, the Heart-of-Mind tradition employs a participatory, competency-based approach to collective regeneration. This model is evident in the intensive training program called Chanmi Xunlian (禅密训练), or “Chan-Esoteric Integrative Training,” observed directly at Damodong and Yuanyin Temples. This program encompasses not only dharani memorization and mudra mastery, but also incorporates contemporary components such as reflective writing, community management, thematic retreats, and public presentation skills. The internal syllabus includes a significant statement: “Future esoteric leaders are not merely memorizersmemorizersmemorizers of mantras, but bridges between inner contemplation, community, and culture.” This marks a shift from a passive liturgical inheritance pattern to an adaptive, multidimensional, and contextual spiritual leadership formation.

This model is producing a new generation of spiritual promoters capable not only of performing rituals but also of being active in the digital realm, conducting cross-border online training, and networking with the Chinese diaspora in Southeast Asia. This approach represents what Gildow and Bingenheimer (2020) call “non-monastic religious professionalism,” where spiritual authority is built not only on lineage but also on social capabilities and community participation. This initiative enables regeneration that is not only vertical (teacher-student), but also horizontal and collective, facilitating the creation of an open, shared learning space oriented towards the sustainability of esoteric values in the context of an increasingly connected and pluralistic world. Heart-of-Mind, therefore, not only preserves ancient teachings but actively shapes a model of religious regeneration that is relevant, inclusive, and participatory in the contemporary landscape of Chinese Buddhism.

2. Social adaptation: contemporary relevance and format transformation

In response to contemporary social realities, particularly for the urban and digital generation, the Heart-of-Mind tradition has adopted a transformative social adaptation strategy. Rather than maintaining the exclusive and closed format of esoteric rites, promoters have repackaged these practices in a more inclusive, contextual, and communicative format. Formerly private esoteric retreats have become “Life Balance Retreats,” featuring modern themes such as digital mindfulness, ecological ethics, and meditation in the workplace. Visual meditation practices are complemented by ambient instrumental music and soft LED projection effects to create an atmosphere that addresses modern spiritual needs. At Damodong Temple, a workshop titled “Wisdom and Technology” (Wisdom and Technology) connects Buddhist wisdom to contemporary issues such as digital addiction, social fatigue, and the attention crisis. These efforts are not merely cosmetic but demonstrate a deep understanding of the psychosocial dynamics of today’s generation. As stated by R11 (a young teacher): “The mantra does not change, but the language must adapt to the times.” (The mantra does not change, but the language must adapt to the times.)

This adaptation fundamentally reflects the process of resemantization, namely the adjustment of meaning without sacrificing substance. In the 2023 edition of the Beginner’s Guide to Meditation pocketbook, it explains: “Esotericism is not something closed; it is an inner language that needs an external bridge.” This statement emphasizes that esoteric practices are no longer positioned as elitist doctrines, but as contemplative paths accessible through culturally and affectively adapted narratives. This transformation aligns with Bingenheimer’s (2022) findings in his study of “ritual repackaging” in contemporary Han Buddhism, which suggests that the success of preserving tradition lies in its ability to cross-translate meanings into new semiotic terrains. Thus, Heart-of-Mind does not simply “modernizemodernizemodernize” rituals, but actively creates a new spiritual space that resonates with social values, life balance, and wisdom that live within the dynamics of the digital world.

3. Legal space and institutional recognition

The Heart-of-Mind tradition has strategically positioned itself within China’s highly regulated legal and religious political landscape. They do not appear as a fringe or anti-authority tradition but as an active partner in a national project emphasising social harmony, patriotism, and religious stability. This is clearly evident in the Annual Report of the Buddhist Association of Jiangsu Province (2021), which identifies Yuanyin and Damodong Temples as “models of esoteric practice that support patriotic values and contribute to community harmony.” This recognition demonstrates that the Heart-of-Mind has successfully aligned its spiritual mission with state policy—a crucial achievement in the context of tight state control over religion. Their collaborative strategy is realized through regular activity reporting, participation in intersect forums, and opening up access to esoteric rites to civil society within a framework of public ethics and national values.

More than just administrative legality, the legitimacy gained by the Heart-of-Mind is symbolic and political. The Yuanyin Temple Charter (2021) explicitly states:

「我們誠心奉行佛法, 亦忠誠於祖國。我們復興儀式, 為的是服務生命, 而非逃避社會。」.

“We are faithful to the Dharma, and also to the motherland. We revive rituals to serve life, not to escape from society.”

This statement reflects a narrative that resonates strongly with the spirit of contemporary Chinese religious nationalism: religious teachings are only valid if they align with national development and do not cause social disruption. By making rituals a form of contribution to “life”—rather than an escape from social reality—Heart-of-Mind has successfully framed itself as an active element in the nation’s civilization. This strategy reinforces the theory of “religious alignment” (Yang, 2021), which explores how minority religious groups survive by aligning their spiritual vision with the dominant state ideology. Heart-of-Mind is a concrete example of how esoteric Buddhism can survive and thrive legally and institutionally amidst the pressures of modernization and strict political control.

## Discussion

### Legitimacy of the heart-of-mind tradition in the context of contemporary Chinese esoteric Buddhism

Findings regarding legitimation strategies in the Heart-of-Mind Tradition indicate that efforts to establish spiritual authority are not solely based on continuity of the guru-disciple lineage, but also through symbolic elaboration, doctrinal affirmation, actual rituals, and strategic institutional legitimacy. This pattern reinforces Max Weber’s thesis on the typology of authority, where legitimacy is established through a combination of charismatic authority (through spiritual figures such as Master Dayu and Master Yuan Yin), traditional authority (through lineage narratives dating back to the Qing Dynasty), and rational-legal authority (through institutional recognition by the Chinese Buddhist Association). This suggests that religious legitimacy in the modern Chinese religious context does not exist in isolation but operates within a hybrid spectrum that bridges doctrine, ritual performativity, and the state’s administrative demands.

The emphasis on the 佛心經 as the centre of spiritual authority is a doctrinal strategy that places the Heart-of-Mind Tradition in continuity with Orthodox Esoteric Buddhism, such as Shingon and Vajrayāna. However, rather than adopting the formalism of empowerment as in the Tibetan or Japanese abhiseka systems, Heart-of-Mind developed confirmation and transmission rites through local formats that still maintain esoteric elements: mantra orality, guided meditation, and silent transmission. This approach indicates a process of adaptation of form without sacrificing essence, as suggested by Orzech et al. (2011); Chen and Heller (2022); and Chen (2024a,b), that the regeneration of modern esoteric Buddhism relies more on epistemological adaptation than mere textual preservation.

Furthermore, legitimacy within this tradition is also reinforced through historical framing and claims to authentic texts as spiritual and cosmological foundations. Quotations from documents such as the Heart Sutra Internal Commentary (佛心乃大日法身智慧之體), which states that “The Buddha-Heart is the embodiment of Mahāvairocana’s Dharmakāya,” imply that this local text is not simply a new development, but rather a reinterpretation of mainstream Tantric Buddhism. In this context, legitimacy is not simply a matter of social acceptance, but also a negotiation with centers of global Buddhist orthodoxy. As Chen (2024a,b) asserts, “esotericism often gains authority not only through textual heritage but also by re-enchanting modern rationality with sacred cosmology,” and Heart-of-Mind makes the text both a pivot for authentication and a tool for resistance to delegitimization.

Institutionally, Heart-of-Mind’s legitimacy has proven to be non-confrontational, but cooperative, with the state’s religious regulatory system. This tradition has successfully articulated esoteric teachings in a narrative compatible with the values of patriotism and social harmony, as reflected in an official document from the Buddhist Association of China (2021). This indicates that legitimacy in the Chinese context is not merely a matter of doctrine or spirituality, but how a tradition is able to frame itself within the language of national morality. Thus, Heart-of-Mind does not merely persist in the spiritual sphere, but is able to shape legal and public spaces that affirm its existence as a legitimate, relevant, and transformative esoteric tradition.

The legitimation strategies found in the Heart-of-Mind Tradition show structural similarities with various forms of revitalized Esoteric Buddhism in East and Southeast Asia, but also possess local specificities that significantly differentiate them. Theoretically, the mechanisms of legitimacy constructed through lineages, authoritative texts, and closed rites bear strong parallels to the patterns of authority discussed by Max Weber and developed by Buddhist scholars such as Chen (2024a,b), who argues that “esotericism maintains legitimacy through regulated secrecy, performative rituality, and the evocation of cosmic alignment.” Within this framework, legitimacy is not simply a product of historical narratives, but the result of a continually negotiated symbolic process.

Empirically, there are parallels with the Neo-Zhenyan revival in China and Taiwan analyzed by Wu (2024), where monks use transnational legitimacy, for example, endorsement from Japanese Shingon priests, to establish new Zhenyan centers in Southeast Asia and the Americas. However, unlike Neo-Zhenyan, which relies heavily on external authority (Japanese Shingon), Heart-of-Mind instead builds its legitimacy from within (endogenous legitimacy), by embracing the Buddha Heart Sutra (佛心經) as its central text and avoiding reliance on foreign orthodox institutions. This demonstrates a hybrid model that is more autonomous yet still esoteric.

### Regeneration and social transmission of esoteric traditions

Findings regarding the role of promoters in the Heart-of-Mind Tradition indicate that the survival of esoteric traditions in China is not solely determined by the power of texts or rituals, but is significantly supported by charismatic figures who act as spiritual conduits and agents of social transformation. Promoters such as Master LZ and Abbot QW emerge as embodiments of charismatic authority within Weber’s (2013) framework, yet with unique local content, where charisma stems not only from spiritual experience but also from the ability to build social networks, public communication skills, and sensitivity to the needs of modern urban communities.

This phenomenon bears similarities to the role of esoteric figures in the Japanese Shingon tradition, such as Kukai (弘法大師), who emphasized not only ritual practice but also intellectual development, public education, and diplomatic relations with state authorities (Matsunaga, 2016). However, unlike Shingon, which developed a hierarchical and exclusive authority system based on formal initiation (abhiseka), Heart-of-Mind developed a collective mentoring model that is participatory, flexible, and inclusive, as demonstrated through the Chanmi Xunlian program and rigorous community documentation practices.

From a comparative perspective, this strategy can also be linked to the phenomenon of esoteric regeneration in Thailand and Vietnam, where local actors such as young ajahns and urban Buddhist figures play a central role in bridging traditional values with the discourse of modernity (Pattharasinsiri, 2019; Pham, 2021). However, Heart-of-Mind is characterized by its active and systematic use of digital media as a channel for esoteric transmission. Online series like “Esoteric Basics” and visual meditation content reflect that promoters think within orthodoxy and act as producers of culture relevant to the digital generation. This aligns with the concept of “ritual technopraxis” (Campbell and Tsuria, 2021), where spiritual teachings are transmitted and modulated through digital formats as a new form of ritualization.

Theoretically, this suggests a synthesis of three Weberian forms of authority, charismatic, traditional, and legal-rational, adapted to the context of Chinese Esoteric Buddhism. Heart-of-mind promoters not only inherited the charisma of their spiritual ancestors but also integrated it with administrative legitimacy through provincial Buddhist associations and constructed new rationalities through documentation practices and media production. Thus, Heart-of-Mind promoters were not only spiritual figures but also “religious technocrats” capable of designing transformative infrastructure for transmitting esoteric teachings in a changing society.

### The sustainability of esoteric traditions within the framework of managed modernity

The findings on the Heart-of-Mind tradition’s sustainability strategies highlight how contemporary esoteric communities maintain vertical spiritual continuity (teacher to student) and develop horizontal regeneration models based on participation, social adaptation, and institutional legitimacy. This demonstrates a significant shift in how contemporary China imagined and practiced esoteric traditions, from a closed and elite heritage to an open, contextual transmission system responsive to societal changes.

The Chanmi Xunlian training model and inclusive formats such as digital mindfulness retreats demonstrate a synthesis between classical teachings and contemporary practices. This strategy is reminiscent of the “reinterpretive transmission” approach in Chen’s (2024a,b) study, in which esoteric texts and rituals are reconfigured to meet the needs of the times without losing their ontological structure. In the Japanese context, a similar approach is found in the Koyasan community’s revitalization of Shingon, which has begun to open public access and revamp its approach to educating young monks (Matsunaga, 2016). The difference is that Heart-of-Mind goes further by designing digital distribution of teachings and strengthening lay involvement as an integral part of regeneration, something rarely done in orthodox esoteric traditions.

Theoretically, this phenomenon reflects two interconnected frameworks. First is the “spiritual reterritorialization” model (Campbell and Tsuria, 2021), in which sacred space is no longer confined to temples or ritual sites but expanded through digital media and modern social spaces. Second, Heart-of-Mind demonstrates the practice of “embedded esotericism” (Asprem and Strube, 2021), in which esoteric traditions do not exist in symbolic isolation but are embedded within the context of environmental ethics, everyday psychology, and religious nationalism. This approach challenges the classical assumption that esotericism always opposes social norms or state control.

From a legal perspective, Heart-of-Mind’s success in gaining recognition from the Provincial Buddhist Association and registering as an official activity centre demonstrates an adaptive capacity to align esoteric teachings with state values such as social harmony and patriotism. This is reminiscent of the concept of “managed religiosity” (Goossaert and Palmer, 2022), in which religious communities in China form a symbiotic relationship with the state to gain operational legitimacy. Heart-of-Mind demonstrates that spiritual sustainability is not merely a doctrinal matter, but also a symbolic politics actively negotiated through narrative and participation.

Thus, Heart-of-Mind’s sustainability is not a passive reproduction of traditions but a complex, regenerative project combining deep spirituality, social skills, technological adaptation, and institutional negotiation. It makes an important contribution to the study of esoteric Buddhism in East Asia by offering a model of sustainability that not only preserves what has been passed down but also creates new possibilities for an inclusive, contextual, and dynamic contemplative future in a modern, pluralistic and interconnected society.

Existing research on the revival strategies of Buddhism in general is insufficient to account for the revival of Esoteric Buddhism because it often overlooks the unique mechanisms through which esoteric traditions establish legitimation, ensure transmission, and maintain continuity in contemporary contexts. While mainstream Buddhist revival studies tend to emphasize institutional reform, state-religion relations, or public engagement, Esoteric Buddhism operates through more complex, often hidden networks of lineage transmission, ritual initiation, and symbolic legitimacy tied to secret doctrines and master-disciple relationships. The Heart-of-Mind Esoteric Buddhist tradition in contemporary China exemplifies this complexity: its revival involves negotiating between ancient tantric practices and modern cultural narratives, balancing spiritual authenticity with social legitimacy, and integrating ritual secrecy within digital and globalized religious environments. Therefore, to fully understand the revival of Esoteric Buddhism, research must move beyond general models of Buddhist modernization and engage with the psycho-cultural, ritual, and transgenerational processes that sustain esoteric continuity amid China’s rapidly transforming religious landscape.

## Conclusion

This study reveals that the Heart-of-Mind Esoteric Buddhism tradition demonstrates the ability to maintain its existence in a manner that is not only conservative but also innovative and contextual. This tradition establishes itself as a legitimate part of China’s religious landscape through complex legitimation strategies, including spiritual transmission lineages, doctrinal justification through sacred texts such as the 佛心經, and integration with the state’s legal framework. Promoters such as charismatic teachers, community leaders, and the younger generation are key to regeneration, maintaining the teachings and aligning them with the dynamics of the times through digital technology, intergenerational training, and down-to-earth narratives. Esoteric practices that were once closed are now being reinterpreted as open, contextual, contemplative spaces responsive to social issues such as urban stress, digital ethics, and life balance. Thus, Heart-of-Mind is not just a surviving sect but an adaptively evolving tradition, shaping a new Buddhist spirituality rooted in classical authority yet functional in modern life. This success underscores the importance of synergy between spiritual authority, social innovation, and contextual awareness as the foundation for the sustainability of religious traditions in a changing society.

## Data Availability

The raw data supporting the conclusions of this article will be made available by the authors, without undue reservation.
